# Rare variants in *SQSTM1* and *VCP* genes and risk of sporadic inclusion body myositis

**DOI:** 10.1016/j.neurobiolaging.2016.07.024

**Published:** 2016-11

**Authors:** Qiang Gang, Conceição Bettencourt, Pedro M. Machado, Stefen Brady, Janice L. Holton, Alan M. Pittman, Deborah Hughes, Estelle Healy, Matthew Parton, David Hilton-Jones, Perry B. Shieh, Merrilee Needham, Christina Liang, Edmar Zanoteli, Leonardo Valente de Camargo, Boel De Paepe, Jan De Bleecker, Aziz Shaibani, Michela Ripolone, Raffaella Violano, Maurizio Moggio, Richard J. Barohn, Mazen M. Dimachkie, Marina Mora, Renato Mantegazza, Simona Zanotti, Andrew B. Singleton, Michael G. Hanna, Henry Houlden, The Muscle Study Group and The International IBM Genetics Consortium, Michael G. Hanna, Henry Houlden, Pedro M. Machado, Qiang Gang, Conceicao Bettencourt, Estelle Healy, Matthew Parton, Janice L. Holton, Stefen Brady, David Hilton-Jones, Perry B. Shieh, Edmar Zanoteli, Leonardo Valente de Camargo, Boel De Paepe, Jan De Bleecker, Aziz Shaibani, Michela Ripolone, Raffaella Violano, Maurizio Moggio, Richard J. Barohn, Mazen M. Dimachkie, April L. McVey, Mamatha Pasnoor, Melanie Glenn, Omar Jawdat, Jeffrey Statland, Gabrielle Rico, Marina Mora, Renato Mantegazza, Simona Zanotti, Merrilee Needham, Frank Mastaglia, Christina Liang, Marinos C. Dalakas, Angie Biba, Hector Chinoy, James B. Lilleker, Janine Lamb, Hazel Platt, Robert G. Cooper, James A.L. Miller, Mark Roberts, Elizabeth Househam, David Hilton, Aditya Shivane, Amy Bartlett, John T. Kissel, Heidi Runk, Matthew Wicklund, David S. Saperstein, Lynette R. McKinney

**Affiliations:** a Department of Molecular Neuroscience, Institute of Neurology, University College London, Queen Square, London, UK; b MRC Centre for Neuromuscular Diseases, Institute of Neurology, University College London, Queen Square, London, UK; c Department of Clinical and Experimental Epilepsy, Institute of Neurology, University College London, Queen Square, London, UK; d Centre for Rheumatology, Division of Medicine, University College London, London, UK; e Nuffield Department of Clinical Neurosciences, John Radcliffe Hospital, Oxford, UK; f Reta Lila Weston Laboratories, UCL Institute of Neurology, London, UK; g Department of Neurology, University of California, Los Angeles, Los Angeles, CA, USA; h Western Australian Neurosciences Research Institute (WANRI), University of Western Australia and Murdoch University, Fiona Stanley Hospital, Perth, Australia; i Department of Neurology, Royal North Shore Hospital, New South Wales, Australia; j Department of Neurology, Medical School of the University of São Paulo (FMUSP), São Paulo, Brazil; k Department of Neurology and Neuromuscular Reference Centre, Ghent University Hospital, Ghent, Belgium; l Nerve and Muscle Center of Texas, Houston, TX, USA; m Neuromuscular Unit, IRCCS Foundation Ca' Granda Ospedale Maggiore Policlinico, Dino Ferrari Centre, University of Milan, Milan, Italy; n The University of Kansas Medical Centre, Kansas City, KS, USA; o Neuromuscular Diseases and Neuroimmunology Unit, Fondazione IRCCS Isitituto Neurologico C. Besta, Milano, Italy; p Laboratory of Neurogenetics, National Institute on Aging, National Institute of Health, Bethesda, MD, USA; q Neurogenetics Laboratory, Institute of Neurology, University College London, Queen Square, London, UK

**Keywords:** Sporadic inclusion body myositis, sIBM, *SQSTM1*, *VCP*, Genetic risk factor

## Abstract

Genetic factors have been suggested to be involved in the pathogenesis of sporadic inclusion body myositis (sIBM). Sequestosome 1 (*SQSTM1*) and valosin-containing protein (*VCP*) are 2 key genes associated with several neurodegenerative disorders but have yet to be thoroughly investigated in sIBM. A candidate gene analysis was conducted using whole-exome sequencing data from 181 sIBM patients, and whole-transcriptome expression analysis was performed in patients with genetic variants of interest. We identified 6 rare missense variants in the *SQSTM1* and *VCP* in 7 sIBM patients (4.0%). Two variants, the *SQSTM1* p.G194R and the *VCP* p.R159C, were significantly overrepresented in this sIBM cohort compared with controls. Five of these variants had been previously reported in patients with degenerative diseases. The messenger RNA levels of major histocompatibility complex genes were upregulated, this elevation being more pronounced in *SQSTM1* patient group. We report for the first time potentially pathogenic *SQSTM1* variants and expand the spectrum of *VCP* variants in sIBM. These data suggest that defects in neurodegenerative pathways may confer genetic susceptibility to sIBM and reinforce the mechanistic overlap in these neurodegenerative disorders.

## 1 Introduction

Sporadic inclusion body myositis (sIBM) is the most common myopathy among people aged >45 years, presenting a characteristic pattern of progressive muscle weakness and atrophy in both proximal and distal muscles, particularly in knee extensors and wrist and finger flexors (Machado et al., 2014). Muscle pathology in sIBM indicates a combination of inflammatory and degenerative features such as rimmed vacuoles, sarcoplasmic inclusions, and the deposition of degenerative proteins in affected muscle as pathologic hallmarks, which are features that differentiate sIBM from other muscle disorders (Machado et al., 2014). Electromyography shows a myopathic and neurogenic pattern in some sIBM patients (Lotz et al., 1989), which resembles some hereditary inclusion body myopathies (hIBMs) and motor neuron diseases (Dabby et al., 2001, Lotz et al., 1989). Genes have been identified as associated with hIBMs, and genetic susceptibility factors could also be involved in the pathogenesis of sIBM. In addition to the known hIBM genes, genes encoding for the proteins abnormally accumulated in sIBM muscle are of great interest (Gang et al., 2014), as many of these proteins, such as amyloid-β, hyperphosphorylated tau, p62, and transactive response (TAR) DNA-binding protein-43 (TDP-43), have also been associated with neurodegenerative diseases including Alzheimer's disease (AD), amyotrophic lateral sclerosis (ALS), and Parkinson's disease. Furthermore, several studies have shown that the major histocompatibility complex (MHC)–related genes are dysregulated in sIBM (Gang et al., 2014). However, no genetic factors have yet been confirmed as associated with sIBM (Gang et al., 2014, Gang et al., 2015).

P62, also known as sequestosome 1 (SQSTM1), has been recognized as a strong biomarker in muscle with a high sensitivity and specificity for sIBM (Brady et al., 2014). Genetic variants in *SQSTM1* had not been investigated in sIBM until a recent study using targeted next-generation sequencing in a group of 79 patients (Weihl et al., 2015). In that study, only a common *SQSTM1* polymorphism was found, unlikely contributing to a rare disease. A recent study reported a splice donor variant in *SQSTM1* in a family with an autosomal dominant distal myopathy and also in an unrelated patient with sporadic distal myopathy (Bucelli et al., 2015). In addition, mutations in *SQSTM1* are well known to be associated with familial and/or sporadic Paget disease of bone, ALS, and frontotemporal dementia (FTD) (Fecto et al., 2011, Kwok et al., 2014, Laurin et al., 2002, Le Ber et al., 2013, Miller et al., 2015, Rubino et al., 2012). Mutations in valosin-containing protein (*VCP*) gene are known to cause an inherited form of IBM with Paget disease and frontotemporal dementia (IBMPFD) (Gidaro et al., 2008, Watts et al., 2004) and have also been reported in cases with ALS and FTD (Johnson et al., 2010, Koppers et al., 2012). Two missense mutations in *VCP* have been recently identified in 2 unrelated IBM patients, one with sIBM and another with family history for late-onset dementia (Weihl et al., 2015).

These findings, along with denervation in muscle electromyography of sIBM patients, suggest a possible genetic overlap between sporadic and IBM-like myopathies and also neurodegenerative diseases. To thoroughly investigate the contribution of *SQSTM1* and *VCP* genes in sIBM, we investigated these 2 genes using whole-exome sequencing data from 181 sIBM patients, which was produced as a part of an International IBM Genetics Consortium.

## 2 Materials and methods

### 2.1 Subjects

This study is part of the International IBM Genetics Consortium, a Muscle Study Group–endorsed project, which currently has members from 17 specialized centers in 7 countries around the world. For this whole-exome sequencing Consortium study, DNA samples from a total number of 181 sIBM patients were collected from 11 centers. Patients diagnosed with sIBM had to have an sIBM diagnosis according to a muscle diseases expert and also had to fulfil the Griggs criteria (Griggs et al., 1995, Tawil and Griggs, 2002), the European Neuromuscular Center 2000 criteria (Badrising et al., 2000), or the MRC 2010 criteria (Hilton-Jones et al., 2010). Neuropathologically healthy controls (*N* = 235) aged >60 years were used as an internal aged control group to compare with our sIBM cohort. The study was approved by the National Research Ethics Service Committee London—Queen Square (research ethics committee reference: 12/LO/1557).

### 2.2 Genetic and bioinformatic analysis

Whole-exome sequencing data were generated for 181 sIBM DNA samples as previously described (Mencacci et al., 2015). In this study, we used a candidate gene approach on these whole-exome sequencing data to investigate variants in *SQSTM1* and *VCP* genes. We excluded all synonymous variants, and all common variants with a population frequency >1% were identified in the 1000 Genomes project (www.1000genomes.org/), in the Exome Variant Server (EVS) database (evs.gs.washington.edu/EVS/), in the Exome Aggregation Consortium (ExAC) Browser (exac.broadinstitute.org/), and in the internal aged controls, as these variants less likely play a role in a rare disease. The filtered variants were confirmed by the conventional Sanger sequencing. The allele frequency of each variant found in sIBM was compared with the ExAC database using Fisher test. The pathogenicity of these variants was evaluated using the following in silico prediction tools: SIFT (Kumar et al., 2009), MutationTaster (Schwarz et al., 2014), and PolyPhen2 (Adzhubei et al., 2010). Genomic evolutionary rate profiling (GERP++) scores were used to estimate the conservation of each variant in multispecies alignments, with higher scores indicating the most conserved nucleotide positions (Davydov et al., 2010).

### 2.3 Messenger RNA expression and real-time quantitative polymerase chain reaction validation

Available flash frozen muscle biopsy tissues from 6 sIBM subjects with variants in *SQSTM1* or *VCP* and 8 controls that were kindly provided by the MRC Sudden Death Brain and Tissue Bank in Edinburgh, UK, were used for the gene expression analysis. Total RNA was isolated from muscle tissue using the miRNeasy kit (Qiagen, Crawley, UK), and the concentration, purity, and integrity of each RNA sample were assessed as previously described (Trabzuni et al., 2011).

Whole-genome expression profiling was performed using the Illumina HumanHT-12 v4 Expression BeadChip (Illumina, Inc, USA) on 3 patients with *SQSTM1* variants (cases 1, 3, and 4), 2 patients with *VCP* variants (cases 6 and 7), and 5 age- and gender-matched controls. Raw expression data were log2 transformed and quantile normalized, and differential expression analysis (patients with *SQSTM1* variants vs. controls and all the patients vs. controls) was performed using the limma Bioconductor package (Ritchie et al., 2015). Genes were considered differentially expressed and used in further analysis, when false discovery rate–adjusted *p* value was <0.05 and absolute log2 fold change was >0.2. Functional enrichment analysis for Gene Ontology terms, KEGG (Kyoto Encyclopedia of Genes and Genomes) pathways, and Human Phenotype Ontology terms was performed using g:Profiler (biit.cs.ut.ee/gprofiler/). Among the upregulated genes, 3 genes associated with inflammation markers (*HLA*-*A*, *CD74*, and *HLA*-*DRA*) were selected for validation using real-time quantitative polymerase chain reaction (RT-qPCR). Briefly, total RNA (600 ng) from the 6 cases and 8 controls was reverse transcribed into complementary DNA using random primers from High-Capacity cDNA Reverse Transcription Kit (Applied Biosystems, USA). Three replicates per sample were assayed for each target gene using Fast SYBR Green PCR Kit (Applied Biosystems) and run in a QuantStudio 6 Flex Real-Time PCR System (Applied Biosystems). Details on primers and RT-qPCR conditions are available on request. Cyclophilin (*PPIA*) was selected as the reference gene. The 2^−ΔΔCt^ method (Schmittgen and Livak, 2008) was used for Ct normalization for each gene and determination of fold changes in gene expression between patients and controls. Mann-Whitney *U* test was performed to analyze the difference of relative gene expression between patient groups and controls. For all the analyses, *p* value <0.05 was considered statistically significant. Statistical analysis was performed using SPSS Statistics 22 (IBM, USA).

## 3 Results

From the entire cohort of 181 sIBM patients, 150 (82.9%) were Caucasians and 16 (8.8%) were from other ethnicities, including Asian Chinese, the Indian subcontinent, and Black Africans (ethnicity information was unavailable for 15 patients). The majority of sIBM patients were male (65.7%). Age of onset, which was collected retrospectively, ranged from 31 to 85 years (mean 59.6 ± 9.6 years). The mean age of the 235 healthy aged controls was 79.1 ± 8.5 years, ranging from 60 to 102 years, and similarly to the sIBM cohort, the majority were also male (61.7%).

**Fig. 1:**
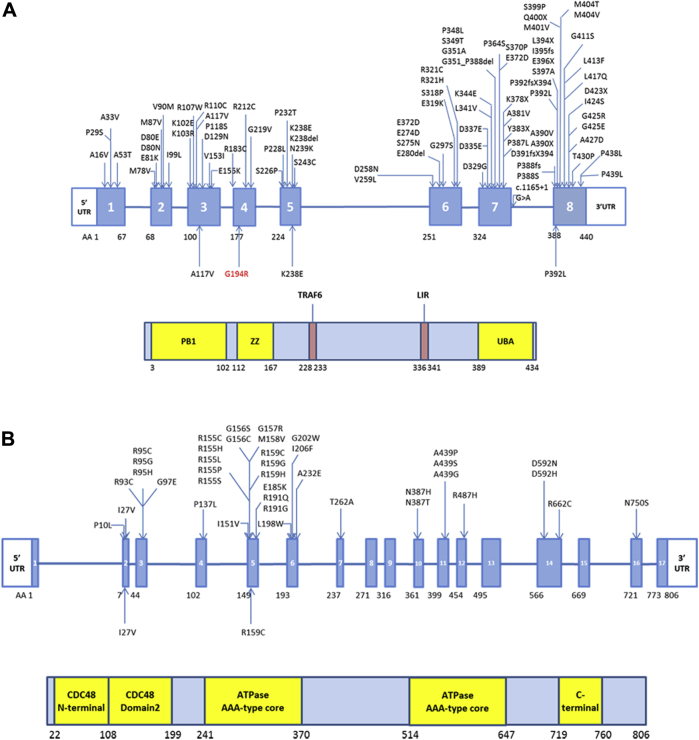
Mutations and/or variants in sequestosome 1 (*SQSTM1*) and valosin-containing protein (*VCP*) genes and their protein domains. (A) Mutations and/or variants found in *SQSTM1* gene and protein domains. (B) Mutations and/or variants found in *VCP* gene and the protein domains. The ones above the gene structure were reported in the previous studies. The ones below were identified in our sporadic inclusion body myositis cohort, and the one labeled in red was not reported in other diseases before. Abbreviations: LIR, LC3-interaction region; PB1, Phox and Bem1p domain; TRAF6, tumor necrosis factor receptor–associated factor 6 binding site; UBA, ubiquitin-associated domain; UTR, untranslated region; ZZ, zinc finger domain.

In this sIBM cohort, 4 rare missense variants in the *SQSTM1* gene (ENST00000389805) were found in 4 patients (Table 1). Two rare missense variants in the *VCP* gene (ENST00000358901) were found in 3 sIBM patients. Of note, the frequency of the variants *SQSTM1* p.G194R and *VCP* p.R159C was significantly higher in our sIBM cohort compared with the ExAC database (Fisher exact test, *p* = 0.018 and *p* = 5.288 × 10^−5^, respectively). These 2 variants were absent in the other population databases and in our aged control group. From the 6 rare variant we found, 4 (Table 1) had been previously reported in patients with ALS (Abramzon et al., 2012, Rubino et al., 2012). Among them, the *SQSTM1* p.P392L is also known to be the most frequent *SQSTM1* mutation in PDB (Laurin et al., 2002) and has also been reported in cases with FTD (Le Ber et al., 2013) and normal tension glaucoma (Scheetz et al., 2016), whereas *VCP* p.I27V and p.R159C have also been found in patients with IBMPFD (Chan et al., 2012, Rohrer et al., 2011), and the *VCP* p.I27V has also been recently reported in one sIBM patient (Weihl et al., 2015). The *SQSTM1* p.A117V was reported in one early-onset AD patient (Cuyvers et al., 2015). Variants found in sIBM patients were absent in our internal aged controls except *SQSTM1* variants previously associated with ALS (p.P392L and p.K238E, Table 1). With the exception of *SQSTM1* p.A117V, all these variants are located at conserved positions among species further suggesting they are functionally relevant. The variants found in our cohort and in previous studies are shown in Fig. 1.

Tables 2 and 3 summarize the demographic, clinical, and muscle biopsy characteristics of the patients carrying *SQSTM1* and *VCP* variants. The 7 sIBM cases fulfilled the MRC 2010 diagnostic category of pathologically defined, clinically defined, or possible sIBM. There was also no family history of muscle diseases, and none of the 7 patients and their families showed evidence of bone or cognitive problems.

**Fig. 2:**
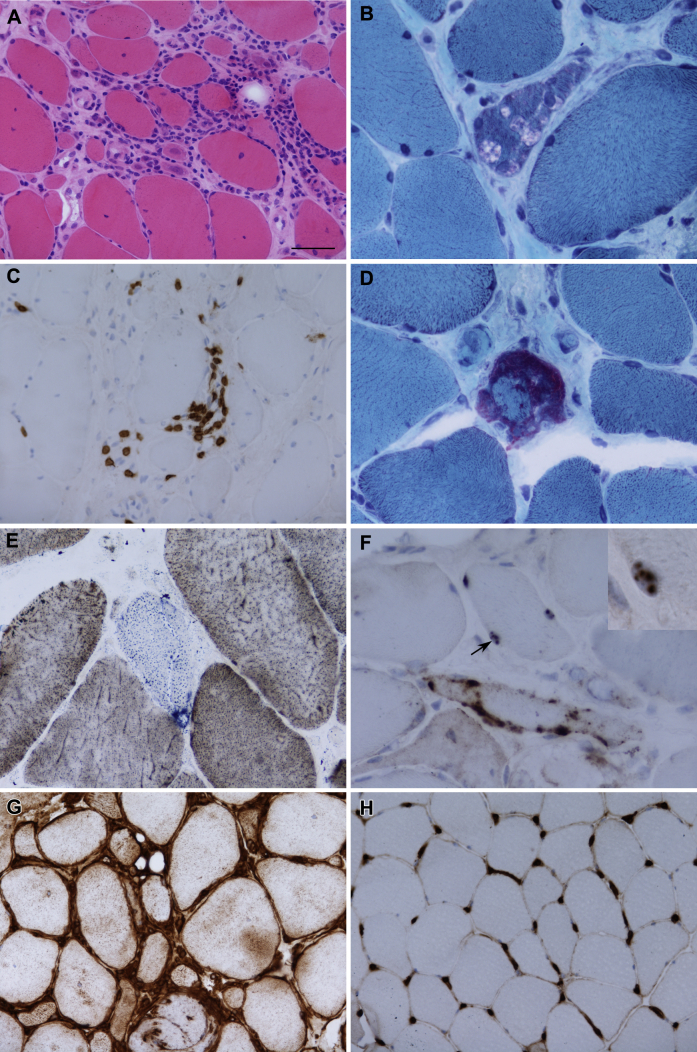
Pathologic features observed in sporadic inclusion body myositis patients. There was variation in fiber size with endomysial inflammation, increased internal nucleation, and fiber regeneration (A, hematoxylin and eosin). Rimmed vacuoles were found in all cases (B, Gomori trichrome). The inflammatory infiltrate contains T lymphocytes (C, CD3). A ragged red fiber was observed in case 2 (D, Gomori trichrome). Cytochrome *c* oxidase negative fibers were identified (E, cytochrome oxidase and/or succinic dehydrogenase). P62 immunoreactive sarcoplasmic inclusions were identified (F) in addition to sparse intranuclear inclusions (arrow and inset). Major histocompatibility complex class I was diffusely increased in patients with sequestosome 1 (*SQSTM1*) variants (G) in comparison with a normal control (H). Scale bar represents 50 μm in A, C, G, and H; 25 μm in B, D, and F; and 10 μm in the inset in F. Panels A and G are from Case 1; panels B–F are from case 2.

Fig. 2 illustrates the pathologic features of muscle biopsies observed in patients carrying variants in *SQSTM1* only because of the availability of the biopsy images. P62-positive inclusions were found in 3 patients with *SQSTM1*. Patients with *SQSTM1* variants showed a global upregulation of MHC-I (diffuse pattern, Fig. 2G) compared with the healthy control (Fig. 2H).

**Fig. 3:**
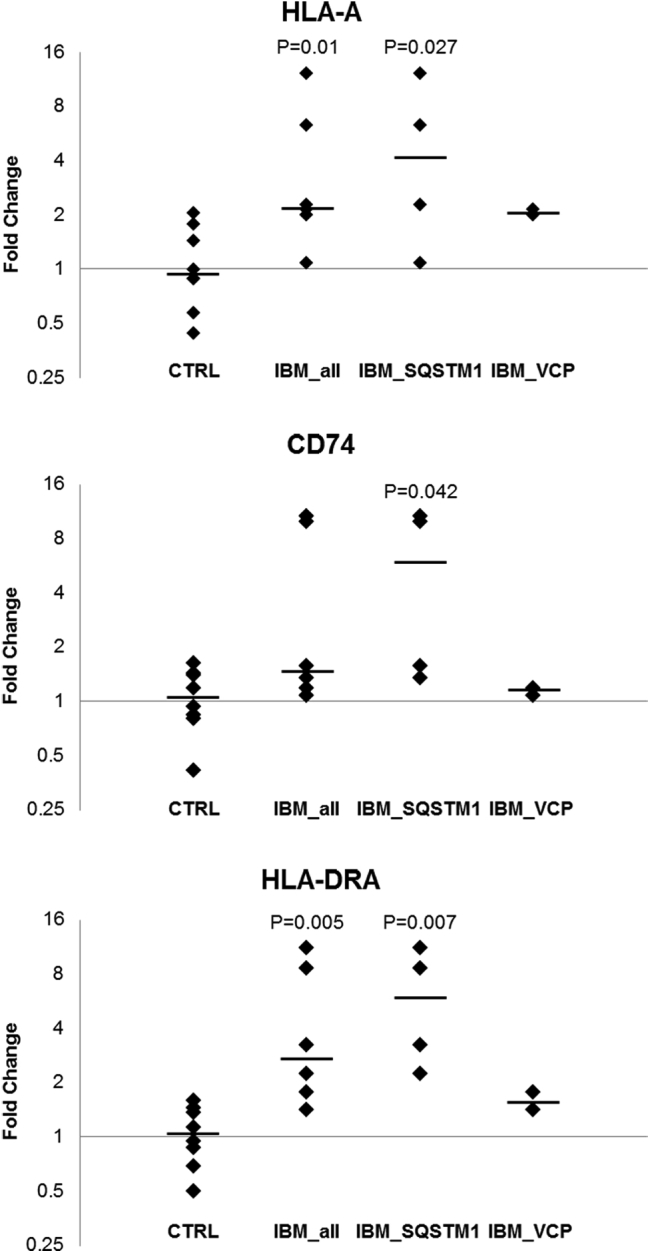
Scatter plot illustrating distribution of the fold change for the expression of 3 major histocompatibility complex genes in sporadic inclusion body myositis groups compared with controls as determined by real-time quantitative polymerase chain reaction. All expression levels were normalized to the expression of the reference gene, *PPIA*, and relative to the mean normalized expression of all the controls. The solid black lines denote the medians. Only significant Mann-Whitney *U* test *p* values (<0.05) regarding comparisons of patient groups with controls are shown. Abbreviations: *CD74*, CD74 molecule, major histocompatibility complex, class II invariant chain; *HLA-A*, major histocompatibility complex, class I, A; *HLA-DRA*, major histocompatibility complex, class II, DR alpha.

To further understand molecular changes occurring in sIBM, particularly those related to the variants in *SQSTM1* and *VCP*, we have performed whole-genome expression analysis. Most of the differential expressed genes were found comparing the *SQSTM1* sIBM patient group with controls (Supplementary Tables 1 and 2), with 33 upregulated and 7 downregulated genes. The small number of available tissue samples (*n* = 2) from patients with *VCP* variants prevented statistical analysis of this sIBM patient group. The expression of *SQSTM1* and *VCP* did not show significant differences between any patient group and controls, but a significant upregulation of MHC genes (class I [*HLA*-*A*] and class II [*CD74* and *HLA*-*DRA*]) was seen in the group of patients carrying *SQSTM1* variants (Supplementary Table 2). RT-qPCR analysis of those MHC genes validated their upregulation in sIBM; this was particularly evident comparing the *SQSTM1* group with the controls, with significant upregulation of all analyzed genes (Fig. 3). Functional enrichment analysis of upregulated genes in the *SQSTM1* patient group showed a significant overrepresentation of several Gene Ontology terms related with immune response, MHC protein complex, and endosome vesicles and KEGG pathways mostly related to inflammatory, autoimmune, and infectious diseases (Supplementary Table 3). The small number of dysregulated genes found in the expression microarray analysis data prevented functional enrichment analysis for other comparison groups.

## 4 Discussion

Using whole-exome sequencing, we identified rare missense variants in the *SQSTM1* and *VCP* genes in 7 sIBM cases. The frequency of patients with rare *SQSTM1* and *VCP* variants in the sIBM cohort was 4.0%. Two independent cases have previously been reported with *VCP* variants (Weihl et al., 2015), but our study extends this finding in a larger cohort of sIBM patients. Regarding *SQSTM1*, this is to our knowledge the first report where possible pathogenic variants in this gene are observed in sIBM patients.

The *SQSTM1* gene encodes for sequestosome 1 and/or p62 (referred as p62 in the article), which is a multifunction protein participating in a number of different biological pathways (Komatsu et al., 2012), including the autophagy pathway and various transduction pathways such as nuclear factor-kappaB signaling and apoptosis. Mutations in *SQSTM1* were first identified in PDB (Laurin et al., 2002), a chronic disease of bone that can cause skeletal deformity and fractures, and account for 25%–50% of familial and 5%–10% of sporadic PDB patients (Ralston and Layfield, 2012). In addition, mutations in *SQSTM1* are also known to contribute to 1%–3.5% of patients with ALS/FTD with or without familial history (Rubino et al., 2012), a similar frequency to the one we found in our sIBM cohort. Mutations in *SQSTM1* are widespread along the gene (Fig. 1A), but the missense mutation p.P392L located in the C-terminal ubiquitin-associated domain, where most mutations lie in, is the most frequent *SQSTM1* mutation in all the different clinical phenotypes (Fecto et al., 2011, Laurin et al., 2002). A mouse model with *sqstm1* p.P394L mutation (Daroszewska et al., 2011), equivalent to *SQSTM1* p.P392L in humans, developed a human PDB-like phenotype and showed dysregulation of autophagy and enhanced autophagosome formation. The *SQSTM1* p.K238E has also been reported in one sporadic ALS (Rubino et al., 2012) and lies in a tumor necrosis factor receptor–associated factor 6 (TRAF6)–binding site—where p62 interacts with TRAF6, a critical component of the nuclear factor-kappaB pathway in response to multifactors, including proinflammatory cytokines (Fecto et al., 2011). The *SQSTM1* p.G194R has not been observed in other diseases and was absent in our aged controls, and it is worth mentioning that it has been found overrepresented our sIBM cohort. Although the *SQSTM1* p.A117V is predicted as benign, it was absent in our aged controls and recently was reported in a patient with early-onset AD (Cuyvers et al., 2015) and thus cannot be excluded as a risk factor for sIBM.

The *VCP* gene encodes for the ATPase valosin-containing protein, which plays a role in proteasomal degradation of misfolded proteins (Meyer and Weihl, 2014). *VCP* is also involved in critical signaling pathways, membrane fusion, cell cycle controls, and more importantly facilitating a cargo sorting via endosomal and/or autophagy pathway (Meyer and Weihl, 2014). Mutations in *VCP* are known to cause IBMPFD (Watts et al., 2004), Parkinson's disease (Majounie et al., 2012), and are also associated with ALS with or without FTD (Johnson et al., 2010). The *VCP* p.I27V variant has been previously reported as potentially pathogenic (Majounie et al., 2012, Rohrer et al., 2011) and was recently found in another patient with sIBM (Weihl et al., 2015). Functional analysis of this variant showed an increase in p62 and LC3II protein levels (Weihl et al., 2015), suggesting it may cause disruption in autophagosome maturation (Ju et al., 2009). The *VCP* p.R159C found to be overrepresented in our sIBM cohort has been previously reported as pathogenic and associated with IBMPFD (Bersano et al., 2009) and sporadic ALS (Abramzon et al., 2012). Two additional mutations were also found at this amino acid residue in familial ALS (p.R159G) (Johnson et al., 2010) and IBMPFD (p.R159H) (Haubenberger et al., 2005). The *VCP* p.R159C lies within the highly conserved CDC48 domain of the protein (Fig. 1B), which is involved in ubiquitin-binding and protein-protein interaction, and a hotspot for *VCP* mutations (Bersano et al., 2009).

We reviewed the clinical and pathologic details of all sIBM patients with *SQSTM1* and *VCP* variants and confirmed that none of them had developed symptoms of PDB, FTD, or ALS and none had family history of these diseases or family history of muscle weakness. VCP staining was not available for all the patients, but p62-positive inclusions were seen in all 3 biopsies where p62 staining was available, including 3 sIBM with *SQSTM1* p.P392L, p.A117V, and p.G194R.

The expression levels of either *SQSTM1* or *VCP* messenger RNA were not significantly altered in patients compared with controls, suggesting that the missense variants found in these patients did not alter the corresponding gene expression at the messenger RNA level. P62 and VCP aggregates in the muscle could be a result of increased protein stability, dysfunction of other factors along the proteasomal or lysosomal pathway, or both (Sandri, 2010).

The MHC class I (*HLA*-*A*) and II (*HLA*-*DRA*) genes were significantly upregulated in patients compared with controls by RT-qPCR, which is consistent with a previous study (Ivanidze et al., 2011), and the other MHC class II gene (*CD74*) was significantly upregulated only in the *SQSTM1* patient group. The statistical analysis could not be carried out for the *VCP* patient group because of small sample size, but there was also a trend for the upregulation of these inflammation markers. The more pronounced upregulation observed in the *SQSTM1* sIBM patient group than in the *VCP* patient group. This is the first time that different expression level of inflammation markers between patients with sIBM is suggested. Although further cases should be analyzed, the MHC expression could be a potential differentiating factor that directs the clinical phenotype of *SQSTM1* or *VCP* toward sIBM as opposed to the other neurodegenerative conditions. Additional analysis in muscle and other tissues of patients carrying *SQSTM1* or *VCP* mutations without symptomatic sIBM but other phenotypes is also necessary in the future to confirm this hypothesis.

In conclusion, we report for the first time likely pathogenic *SQSTM1* variants and expand the spectrum of *VCP* variants in sIBM. Our findings suggest that variants in these genes constitute genetic susceptibility factors for sIBM and for other multisystem proteinopathy phenotypes. The findings from this study also expand the clinicopathologic spectrum of diseases associated with *SQSTM1* and *VCP* genes, and the overlap between sIBM and IBMPFD, ALS, and/or FTD suggests that muscle and brain diseases share similar pathogenic pathways that may be important for further biomarkers, genes, and therapeutic target discovery. Further investigation of the sIBM whole-exome sequencing data is still ongoing and data from this international collaboration will likely reveal further findings.

## Disclosure statement

Declaration of interests: All authors have no competing financial interests. Authors' contributions: Qiang Gang contributed to sample collection, all the experimental work, data analysis, and drafting the first version of the manuscript; Conceição Bettencourt contributed to the experimental plans of microarray and RT-qPCR, preliminary analysis of microarray data, and statistical plan; Pedro M. Machado contributed to overall coordination of the study, namely worldwide sample collection and liaising with all the study collaborators; Conceição Bettencourt, Pedro M. Machado, and Henry Houlden also contributed to drafting the first version of the manuscript; Janice L. Holton contributed to the review and photography of the muscle biopsies; Alan M. Pittman and Deborah Hughes contributed to the generation of whole-exome sequencing data; Stefen Brady, Janice L. Holton, and Boel De Paepe contributed to immunotyping of muscle biopsies; and Andrew B. Singleton contributed to the exome data from control individuals. All the authors from International IBM Genetic Consortium and Muscle Study Group contributed to acquisition of clinical data and sample collection. Michael G. Hanna, Henry Houlden, and Pedro M. Machado are principal investigators of the International IBM Consortium Genetics Study. All the authors contributed to the critical revision of the manuscript and approved the final version.

## Figures and Tables

**Table 1:** *SQSTM1* and *VCP* rare genetic variants in patients with sIBM

Case ID	Gene and region	Variants (heterozygous)	MAF in 235 neuropathologic controls (%)	MAF in 1000 genomes (%)	MAF in ExAC (%)	MAF in sIBM cohort (%)	GERP++ score	PolyPhen prediction	Known in other diseases
Case 1 (sIBM)	*SQSTM1* Exon 8	p.P392L (rs104893941)	0.213	0.46	0.089	0.275	4.43	Pathogenic	Familial PDB and ALS
Case 2 (sIBM)	*SQSTM1* Exon 3	p.A117V (rs147810437)	0	0.18	0.152	0.275	−5.17	Benign	Early-onset AD
Case 3 (sIBM)	*SQSTM1* Exon 4	p.G194R	0	—	0.0017	0.275	3.65	Possibly damaging	—
Case 4 (sIBM)	*SQSTM1* Exon 5	p.K238E (rs11548633)	0.638	0.32	0.242	0.275	3.87	Possibly damaging	ALS
Case 5 (sIBM)	*VCP* Exon 2	p.I27V (rs140913250)	0	0.09	0.054	0.275	5.71	Benign	IBMPFD, ALS, and PD
Case 6 (sIBM) Case7 (sIBM)	*VCP* Exon 5	p.R159C	0	—	0.00082	0.549	4.62	Possibly damaging	IBMPFD, IBM with PD, and sporadic ALS

Key: A, alanine; AD, Alzheimer's disease; ALS, amyotrophic lateral sclerosis; C, cysteine; E, glutamic acid; ExAC, Exome Aggregation Consortium; G, glycine; GERP, genomic evolutionary rate profiling; I, isoleucine; IBMPFD, inclusion body myopathy with Paget disease and frontotemporal dementia; K, lysine; L, leucine; MAF, minor allele frequency; P, proline; PD, Parkinson's disease; PDB, Paget disease of bone; R, arginine; sIBM, sporadic inclusion body myositis; *SQSTM1*, sequestosome 1; V, valine; *VCP*, valosin-containing protein.

**Table 2:** Demographic and clinical features of sIBM patients carrying variants in *SQSTM1* and *VCP* genes

Features	Case 1	Case 2	Case 3	Case 4	Case 5	Case 6	Case 7
Sex	F	M	M	F	M	F	M
Ethnicity	Caucasian	Caucasian	Indian subcontinent	Caucasian	Caucasian	Caucasian	Caucasian
Age at onset	45	50	71	57	85	74	48
Family history	−	−	−	−	−	−	−
Finger flexor weakness	+	+	+	+	+	+	+
Weakness of KE > HF	−	−	+	+	UNK	−	−
Weakness of FF > SA	+	+	+	+	UNK	+	+
Weakness of WF > WE	+	+	+	−	UNK	−	−
PDB	−	−	−	−	−	−	−
ALS	−	−	−	−	−	−	−
FTD	−	−	−	−	−	−	−
Parkinson's disease	−	−	−	−	−	−	−
Elevated CK (×ULN)	+ (≤15)	+ (≤15)	+ (≤15)	−	−	N/A	+ (≤15)
Neurophysiological investigation	Myopathic	Myopathic	Myopathic	N/A	UNK	N/A	N/A
MRC 2010 sIBM diagnostic category	PAD	PAD	PAD	CLD	PO	PO	PO

Key: ALS, amyotrophic lateral sclerosis; CK, creatine kinase; CLD, clinically defined; F, female; FF, finger flexion; FTD, frontotemporal dementia; HF, hip flexion; KE, knee extension; M, male; N/A, not available; PAD, pathologically defined; PDB, Paget disease of bone; PO, possible; SA, shoulder abduction; sIBM, sporadic inclusion body myositis; *SQSTM1*, sequestosome 1; ULN, upper limit of normal; UNK, unknown; *VCP*, valosin-containing protein; WE, wrist extension; WF, wrist flexion.

**Table 3:** Main muscle biopsy features of sIBM patients carrying variants in *SQSTM1* and *VCP* genes

Muscle biopsy features	Case 1	Case 2	Case 3	Case 4	Case 5	Case 6	Case 7
Endomysial exudate	+	+	+	+	+	+	+
MHC-I upregulation	+	+	+	+	+	+	+
Partial invasion	+	+	+	−	+	−	−
Rimmed vacuoles	+	+	+	+	+	−	−
p62 (sarcoplasmic and intranuclear inclusions)	+	+	+	N/A	N/A	N/A	N/A
TDP-43	N/A	+	N/A	N/A	N/A	N/A	N/A
15–18 (or 16–21) nm filaments	N/A	N/A	N/A	−	N/A	N/A	N/A
COX-deficient fibers	+	+	−	+	−	+	+

Key: COX, cytochrome *c* oxidase; MHC, major histocompatibility complex; N/A, not available; sIBM, sporadic inclusion body myositis; *SQSTM1*, sequestosome 1; TDP-43, TAR DNA-binding protein-43; *VCP*, valosin-containing protein.
