# Supplementary material for: Rare variants in SQSTM1 and VCP genes and risk of sporadic inclusion body myositis
Source: Neurobiol Aging. 2016 Nov;47:218.e1–9. doi: 10.1016/j.neurobiolaging.2016.07.024 (PMC5082791; doi:10.1016/j.neurobiolaging.2016.07.024)
Supplement: Supplementary Tables 1–3 [file mmc1.docx]

**Supplementary Table 1. Up/down-regulated genes in the microarray analysis of all the sIBM vs controls.**

| **PROBE_ID** | **SYMBOL** | **DEFINITION** | **logFC** | **P.Value** | **adj.P.Val** |
| --- | --- | --- | --- | --- | --- |
| ILMN_1700316 | *LOC440055* | PREDICTED: Homo sapiens similar to ribosomal protein S12 (LOC440055), mRNA. | 0.265176 | 1.20E-05 | 0.023722 |
| ILMN_1704315 | *LOC389435* | Homo sapiens hCG21078 (LOC389435), mRNA. | 0.570352 | 1.77E-05 | 0.023722 |
| ILMN_2116366 | *RPL12* | Homo sapiens ribosomal protein L12 (RPL12), mRNA. | 0.396815 | 3.66E-05 | 0.036006 |
| ILMN_1685722 | *EIF4A2* | Homo sapiens eukaryotic translation initiation factor 4A, isoform 2 (EIF4A2), mRNA. | -0.42772 | 9.79E-05 | 0.047704 |

*Given the small number of dysregulated genes in the overall analysis, no functional enrichment analysis was performed.

**Supplementary Table 2. Up/down-regulated genes in the microarray analysis of the *SQSTM1* sIBM patient group vs controls.**

| **PROBE_ID** | **SYMBOL** | **DEFINITION** | **logFC** | **P.Value** | **adj.P.Val** |
| --- | --- | --- | --- | --- | --- |
| ILMN_2203950 | *HLA-A* | Homo sapiens major histocompatibility complex, class I, A (HLA-A), mRNA. | 1.697545 | 8.21E-09 | 5.93E-05 |
| ILMN_1800354 | *CST3* | Homo sapiens cystatin C (CST3), mRNA. | 1.345043 | 5.28E-06 | 0.003326 |
| ILMN_1736567 | *CD74* | Homo sapiens CD74 molecule, major histocompatibility complex, class II invariant chain (CD74), transcript variant 1, mRNA. | 1.33614 | 4.33E-06 | 0.002934 |
| ILMN_1725427 | *B2M* | Homo sapiens beta-2-microglobulin (B2M), mRNA. | 1.070304 | 1.40E-08 | 7.60E-05 |
| ILMN_2152131 | *ACTB* | Homo sapiens actin, beta (ACTB), mRNA. | 0.999226 | 0.000573 | 0.028821 |
| ILMN_2038777 | *ACTB* | Homo sapiens actin, beta (ACTB), mRNA. | 0.982321 | 0.000867 | 0.035755 |
| ILMN_1343291 | *EEF1A1* | Homo sapiens eukaryotic translation elongation factor 1 alpha 1 (EEF1A1), mRNA. | 0.934575 | 0.00017 | 0.015975 |
| ILMN_2157441 | *HLA-DRA* | Homo sapiens major histocompatibility complex, class II, DR alpha (HLA-DRA), mRNA. | 0.918773 | 0.0002 | 0.016682 |
| ILMN_1812392 | *TMSB10* | Homo sapiens thymosin beta 10 (TMSB10), mRNA. | 0.897223 | 0.001149 | 0.041167 |
| ILMN_1683271 | *TMSB4X* | Homo sapiens thymosin, beta 4, X-linked (TMSB4X), mRNA. | 0.860586 | 0.000178 | 0.016206 |
| ILMN_2130441 | *HLA-H* | Homo sapiens major histocompatibility complex, class I, H (pseudogene) (HLA-H), non-coding RNA. | 0.730665 | 7.95E-06 | 0.003728 |
| ILMN_2379644 | *CD74* | Homo sapiens CD74 molecule, major histocompatibility complex, class II invariant chain (CD74), transcript variant 2, mRNA. | 0.704286 | 0.000201 | 0.016701 |
| ILMN_3256742 | *LOC100129902* | PREDICTED: Homo sapiens similar to mCG7602 (LOC100129902), mRNA. | 0.676803 | 0.001328 | 0.04352 |
| ILMN_1778401 | *HLA-B* | Homo sapiens major histocompatibility complex, class I, B (HLA-B), mRNA. | 0.664872 | 3.43E-05 | 0.007274 |
| ILMN_1704315 | *LOC389435* | Homo sapiens hCG21078 (LOC389435), mRNA. | 0.613575 | 5.44E-05 | 0.009308 |
| ILMN_1689655 | *HLA-DRA* | Homo sapiens major histocompatibility complex, class II, DR alpha (HLA-DRA), mRNA. | 0.611838 | 0.00037 | 0.022843 |
| ILMN_2148459 | *B2M* | Homo sapiens beta-2-microglobulin (B2M), mRNA. | 0.567881 | 0.000111 | 0.0124 |
| ILMN_1713086 | *RPL27A* | Homo sapiens ribosomal protein L27a (RPL27A), mRNA. | 0.565704 | 0.000731 | 0.032647 |
| ILMN_2046730 | *S100A10* | Homo sapiens S100 calcium binding protein A10 (S100A10), mRNA. | 0.531182 | 0.001396 | 0.044466 |
| ILMN_1765258 | *HLA-E* | Homo sapiens major histocompatibility complex, class I, E (HLA-E), mRNA. | 0.463181 | 0.001422 | 0.044605 |
| ILMN_1772218 | *HLA-DPA1* | Homo sapiens major histocompatibility complex, class II, DP alpha 1 (HLA-DPA1), mRNA. | 0.452916 | 0.000121 | 0.012932 |
| ILMN_2393765 | *IGLL1* | Homo sapiens immunoglobulin lambda-like polypeptide 1 (IGLL1), transcript variant 1, mRNA. | 0.433705 | 9.56E-05 | 0.011618 |
| ILMN_1752592 | *HLA-DRB4* | Homo sapiens major histocompatibility complex, class II, DR beta 4 (HLA-DRB4), mRNA. | 0.416486 | 9.82E-05 | 0.011618 |
| ILMN_2116366 | *RPL12* | Homo sapiens ribosomal protein L12 (RPL12), mRNA. | 0.416108 | 0.000174 | 0.016128 |
| ILMN_3228688 | *LOC730415* | PREDICTED: Homo sapiens hypothetical LOC730415, transcript variant 2 (LOC730415), mRNA. | 0.403135 | 5.44E-05 | 0.009308 |
| ILMN_1684306 | *S100A4* | Homo sapiens S100 calcium binding protein A4 (S100A4), transcript variant 2, mRNA. | 0.394127 | 0.000976 | 0.03811 |
| ILMN_2058782 | *IFI27* | Homo sapiens interferon, alpha-inducible protein 27 (IFI27), transcript variant 2, mRNA. | 0.381146 | 3.56E-06 | 0.002658 |
| ILMN_2409167 | *ANXA2* | Homo sapiens annexin A2 (ANXA2), transcript variant 1, mRNA. | 0.333895 | 0.001462 | 0.045365 |
| ILMN_2053178 | *ACTG1* | Homo sapiens actin, gamma 1 (ACTG1), mRNA. | 0.333156 | 0.00131 | 0.043378 |
| ILMN_2148819 | *TUBA1A* | Homo sapiens tubulin, alpha 1a (TUBA1A), mRNA. | 0.30505 | 4.54E-05 | 0.00845 |
| ILMN_1796712 | *S100A10* | Homo sapiens S100 calcium binding protein A10 (annexin II ligand, calpactin I, light polypeptide (p11)) (S100A10), mRNA. | 0.277185 | 0.001248 | 0.042185 |
| ILMN_1806017 | *PSME1* | Homo sapiens proteasome (prosome, macropain) activator subunit 1 (PA28 alpha) (PSME1), transcript variant 1, mRNA. | 0.269909 | 0.000101 | 0.011694 |
| ILMN_1700316 | *LOC440055* | PREDICTED: Homo sapiens similar to ribosomal protein S12 (LOC440055), mRNA. | 0.255721 | 0.000123 | 0.012932 |
| ILMN_2189936 | *RPL36AL* | Homo sapiens ribosomal protein L36a-like (RPL36AL), mRNA. | 0.242778 | 0.000293 | 0.020117 |
| ILMN_1716678 | *NPC2* | Homo sapiens Niemann-Pick disease, type C2 (NPC2), mRNA. | 0.216139 | 4.41E-05 | 0.008414 |
| ILMN_1810577 | *RPS4X* | Homo sapiens ribosomal protein S4, X-linked (RPS4X), mRNA. | 0.212198 | 0.000265 | 0.01925 |
| ILMN_2038776 | *TXN* | Homo sapiens thioredoxin (TXN), mRNA. | 0.209643 | 1.27E-06 | 0.001451 |
| ILMN_3241758 | *POTEF* | Homo sapiens POTE ankyrin domain family, member F (POTEF), mRNA. | 0.207325 | 0.001137 | 0.040977 |
| ILMN_1794595 | *GAMT* | Homo sapiens guanidinoacetate N-methyltransferase (GAMT), transcript variant 1, mRNA. | -0.28579 | 0.001056 | 0.039317 |
| ILMN_2371825 | *AGL* | Homo sapiens amylo-1, 6-glucosidase, 4-alpha-glucanotransferase (AGL), transcript variant 5, mRNA. | -0.29323 | 0.001208 | 0.041646 |
| ILMN_1738383 | *EEF2* | Homo sapiens eukaryotic translation elongation factor 2 (EEF2), mRNA. | -0.31977 | 0.001135 | 0.040962 |
| ILMN_1687971 | *CAPN3* | Homo sapiens calpain 3, (p94) (CAPN3), transcript variant 2, mRNA. | -0.45792 | 0.001588 | 0.046861 |
| ILMN_1685722 | *EIF4A2* | Homo sapiens eukaryotic translation initiation factor 4A, isoform 2 (EIF4A2), mRNA. | -0.45877 | 0.000314 | 0.021052 |
| ILMN_1756469 | *GAMT* | Homo sapiens guanidinoacetate N-methyltransferase (GAMT), transcript variant 1, mRNA. | -0.49518 | 0.000475 | 0.026259 |
| ILMN_1769191 | *GNAS* | Homo sapiens GNAS complex locus (GNAS), transcript variant 4, mRNA. | -0.56318 | 0.000495 | 0.026739 |
| ILMN_1789950 | *C20orf166* | Homo sapiens chromosome 20 open reading frame 166 (C20orf166), mRNA. | -0.56343 | 0.000102 | 0.01172 |

*Given the small number of down-regulated genes, only the up-regulated genes from this table were used for functional enrichment analysis.

**Supplementary Table 3. Significantly enriched terms obtained by functional enrichment analysis of dysregulated genes in the *SQSTM1* sIBM patient group vs controls comparisons^a^.**

| **TERM ID** | **TERM TYPE** | **TERM GROUP** | **TERM NAME** | **GENE LIST** | **P-VALUE** |
| --- | --- | --- | --- | --- | --- |
| **Up-regulated genes selected for functional enrichment analysis** | | | | | |
| GO:0001895 | BP | 13 | retina homeostasis | *ACTB,B2M,ACTG1,POTEF* | 0.00965 |
| GO:0001906 | BP | 11 | cell killing | *B2M,HLA-E,HLA-A,HLA-B* | 0.0419 |
| GO:0002376 | BP | 11 | immune system process | *CD74,ACTB,PSME1,IGLL1,TXN,IFI27,B2M,ANXA2,ACTG1,HLA-DRA,HLA-E,HLA-H,HLA-A,HLA-DPA1,HLA-B* | 0.00094 |
| GO:0019882 | BP | 11 | antigen processing and presentation | *CD74,PSME1,B2M,HLA-DRA,HLA-E,HLA-H,HLA-A,HLA-DPA1,HLA-B* | 8.78E-08 |
| GO:0048002 | BP | 11 | antigen processing and presentation of peptide antigen | *CD74,PSME1,B2M,HLA-DRA,HLA-E,HLA-H,HLA-A,HLA-DPA1,HLA-B* | 1.28E-08 |
| GO:0002428 | BP | 11 | antigen processing and presentation of peptide antigen via MHC class Ib | *B2M,HLA-E* | 0.0498 |
| GO:0002474 | BP | 11 | antigen processing and presentation of peptide antigen via MHC class I | *PSME1,B2M,HLA-E,HLA-H,HLA-A,HLA-B* | 2.01E-05 |
| GO:0019883 | BP | 11 | antigen processing and presentation of endogenous antigen | *CD74,B2M,HLA-E,HLA-A,HLA-B* | 3.38E-08 |
| GO:0002483 | BP | 11 | antigen processing and presentation of endogenous peptide antigen | *B2M,HLA-E,HLA-A,HLA-B* | 6.38E-06 |
| GO:0019885 | BP | 11 | antigen processing and presentation of endogenous peptide antigen via MHC class I | *B2M,HLA-A,HLA-B* | 0.00149 |
| GO:0019884 | BP | 11 | antigen processing and presentation of exogenous antigen | *CD74,PSME1,B2M,HLA-DRA,HLA-E,HLA-A,HLA-DPA1,HLA-B* | 3.36E-07 |
| GO:0002478 | BP | 11 | antigen processing and presentation of exogenous peptide antigen | *CD74,PSME1,B2M,HLA-DRA,HLA-E,HLA-A,HLA-DPA1,HLA-B* | 2.44E-07 |
| GO:0042590 | BP | 11 | antigen processing and presentation of exogenous peptide antigen via MHC class I | *PSME1,B2M,HLA-E,HLA-A,HLA-B* | 0.00027 |
| GO:0002479 | BP | 11 | antigen processing and presentation of exogenous peptide antigen via MHC class I, TAP-dependent | *PSME1,B2M,HLA-E,HLA-A,HLA-B* | 0.000209 |
| GO:0002480 | BP | 11 | antigen processing and presentation of exogenous peptide antigen via MHC class I, TAP-independent | *B2M,HLA-E,HLA-A,HLA-B* | 1.13E-06 |
| GO:0002449 | BP | 11 | lymphocyte mediated immunity | *CD74,B2M,HLA-E,HLA-A,HLA-B* | 0.0384 |
| GO:0031341 | BP | 11 | regulation of cell killing | *B2M,HLA-E,HLA-A,HLA-B* | 0.00314 |
| GO:0031342 | BP | 11 | negative regulation of cell killing | *HLA-E,HLA-A,HLA-B* | 0.00307 |
| GO:0002700 | BP | 11 | regulation of production of molecular mediator of immune response | *CD74,B2M,HLA-E,HLA-A* | 0.0296 |
| GO:0002706 | BP | 11 | regulation of lymphocyte mediated immunity | *B2M,HLA-E,HLA-A,HLA-B* | 0.0403 |
| GO:0002707 | BP | 11 | negative regulation of lymphocyte mediated immunity | *HLA-E,HLA-A,HLA-B* | 0.0362 |
| GO:0006955 | BP | 11 | immune response | *CD74,ACTB,PSME1,IGLL1,TXN,IFI27,B2M,ACTG1,HLA-DRA,HLA-E,HLA-H,HLA-A,HLA-DPA1,HLA-B* | 2.13E-05 |
| GO:0006952 | BP | 11 | defense response | *CD74,ACTB,PSME1,CST3,TXN,IFI27,B2M,ACTG1,HLA-DRA,HLA-E,HLA-A,HLA-DPA1,HLA-B* | 0.000647 |
| GO:0045087 | BP | 11 | innate immune response | *ACTB,PSME1,TXN,IFI27,B2M,ACTG1,HLA-DRA,HLA-E,HLA-A,HLA-DPA1,HLA-B* | 0.000238 |
| GO:0048584 | BP | 11 | positive regulation of response to stimulus | *CD74,ACTB,PSME1,TXN,B2M,ACTG1,S100A4,HLA-DRA,HLA-E,HLA-A,HLA-DPA1,HLA-B* | 0.0285 |
| GO:0034097 | BP | 11 | response to cytokine | *CD74,IFI27,B2M,HLA-DRA,HLA-E,HLA-A,HLA-DPA1,HLA-B* | 0.0141 |
| GO:0071345 | BP | 11 | cellular response to cytokine stimulus | *CD74,IFI27,B2M,HLA-DRA,HLA-E,HLA-A,HLA-DPA1,HLA-B* | 0.0046 |
| GO:0034341 | BP | 11 | response to interferon-gamma | *B2M,HLA-DRA,HLA-E,HLA-A,HLA-DPA1,HLA-B* | 0.0002 |
| GO:0071346 | BP | 11 | cellular response to interferon-gamma | *B2M,HLA-DRA,HLA-E,HLA-A,HLA-DPA1,HLA-B* | 9.11E-05 |
| GO:0034340 | BP | 11 | response to type I interferon | *IFI27,HLA-E,HLA-A,HLA-B* | 0.0146 |
| GO:0071357 | BP | 11 | cellular response to type I interferon | *IFI27,HLA-E,HLA-A,HLA-B* | 0.0139 |
| GO:0002682 | BP | 11 | regulation of immune system process | *CD74,ACTB,PSME1,B2M,ACTG1,HLA-DRA,HLA-E,HLA-A,HLA-DPA1,HLA-B* | 0.043 |
| GO:0002684 | BP | 11 | positive regulation of immune system process | *CD74,ACTB,PSME1,B2M,ACTG1,HLA-DRA,HLA-E,HLA-A,HLA-DPA1,HLA-B* | 0.000873 |
| GO:0002699 | BP | 11 | positive regulation of immune effector process | *CD74,B2M,HLA-E,HLA-A,HLA-B* | 0.00582 |
| GO:0002705 | BP | 11 | positive regulation of leukocyte mediated immunity | *B2M,HLA-E,HLA-A,HLA-B* | 0.0168 |
| GO:0002708 | BP | 11 | positive regulation of lymphocyte mediated immunity | *B2M,HLA-E,HLA-A,HLA-B* | 0.00729 |
| GO:0002702 | BP | 11 | positive regulation of production of molecular mediator of immune response | *CD74,B2M,HLA-E,HLA-A* | 0.00505 |
| GO:0050776 | BP | 11 | regulation of immune response | *CD74,ACTB,PSME1,B2M,ACTG1,HLA-DRA,HLA-E,HLA-A,HLA-DPA1,HLA-B* | 0.000749 |
| GO:0050778 | BP | 11 | positive regulation of immune response | *CD74,ACTB,PSME1,B2M,ACTG1,HLA-DRA,HLA-E,HLA-A,HLA-DPA1,HLA-B* | 3.97E-05 |
| GO:0002821 | BP | 11 | positive regulation of adaptive immune response | *B2M,HLA-E,HLA-A,HLA-B* | 0.00818 |
| GO:0002460 | BP | 11 | adaptive immune response based on somatic recombination of immune receptors built from immunoglobulin superfamily domains | *CD74,B2M,HLA-E,HLA-A,HLA-B* | 0.036 |
| GO:0002822 | BP | 11 | regulation of adaptive immune response based on somatic recombination of immune receptors built from immunoglobulin superfamily domains | *B2M,HLA-E,HLA-A,HLA-B* | 0.0389 |
| GO:0002824 | BP | 11 | positive regulation of adaptive immune response based on somatic recombination of immune receptors built from immunoglobulin superfamily domains | *B2M,HLA-E,HLA-A,HLA-B* | 0.00687 |
| GO:0002456 | BP | 11 | T cell mediated immunity | *B2M,HLA-E,HLA-A,HLA-B* | 0.00818 |
| GO:0002709 | BP | 11 | regulation of T cell mediated immunity | *B2M,HLA-E,HLA-A,HLA-B* | 0.00168 |
| GO:0002711 | BP | 11 | positive regulation of T cell mediated immunity | *B2M,HLA-E,HLA-A,HLA-B* | 0.000315 |
| GO:0001909 | BP | 11 | leukocyte mediated cytotoxicity | *B2M,HLA-E,HLA-A,HLA-B* | 0.0146 |
| GO:0001910 | BP | 11 | regulation of leukocyte mediated cytotoxicity | *B2M,HLA-E,HLA-A,HLA-B* | 0.00182 |
| GO:0001911 | BP | 11 | negative regulation of leukocyte mediated cytotoxicity | *HLA-E,HLA-A,HLA-B* | 0.00194 |
| GO:0001913 | BP | 11 | T cell mediated cytotoxicity | *B2M,HLA-E,HLA-A,HLA-B* | 0.000209 |
| GO:0001914 | BP | 11 | regulation of T cell mediated cytotoxicity | *B2M,HLA-E,HLA-A,HLA-B* | 0.000053 |
| GO:0031343 | BP | 11 | positive regulation of cell killing | *B2M,HLA-E,HLA-A,HLA-B* | 0.000878 |
| GO:0001912 | BP | 11 | positive regulation of leukocyte mediated cytotoxicity | *B2M,HLA-E,HLA-A,HLA-B* | 0.000514 |
| GO:0001916 | BP | 11 | positive regulation of T cell mediated cytotoxicity | *B2M,HLA-E,HLA-A,HLA-B* | 2.12E-05 |
| GO:0019221 | BP | 11 | cytokine-mediated signaling pathway | *CD74,IFI27,B2M,HLA-DRA,HLA-E,HLA-A,HLA-DPA1,HLA-B* | 0.000668 |
| GO:0060337 | BP | 11 | type I interferon signaling pathway | *IFI27,HLA-E,HLA-A,HLA-B* | 0.0139 |
| GO:0060333 | BP | 11 | interferon-gamma-mediated signaling pathway | *B2M,HLA-DRA,HLA-E,HLA-A,HLA-DPA1,HLA-B* | 5.58E-06 |
| GO:0002715 | BP | 11 | regulation of natural killer cell mediated immunity | *HLA-E,HLA-A,HLA-B* | 0.0362 |
| GO:0002716 | BP | 11 | negative regulation of natural killer cell mediated immunity | *HLA-E,HLA-A,HLA-B* | 0.000815 |
| GO:0002720 | BP | 11 | positive regulation of cytokine production involved in immune response | *CD74,B2M,HLA-A* | 0.033 |
| GO:0042269 | BP | 11 | regulation of natural killer cell mediated cytotoxicity | *HLA-E,HLA-A,HLA-B* | 0.033 |
| GO:0045953 | BP | 11 | negative regulation of natural killer cell mediated cytotoxicity | *HLA-E,HLA-A,HLA-B* | 0.000815 |
| GO:0042270 | BP | 11 | protection from natural killer cell mediated cytotoxicity | *HLA-E,HLA-A,HLA-B* | 0.000136 |
| GO:0001775 | BP | 5 | cell activation | *CD74,ACTB,CST3,B2M,ACTG1,HLA-DRA,HLA-E,TMSB4X,HLA-A,HLA-DPA1* | 0.00101 |
| GO:0035740 | BP | 37 | CD8-positive, alpha-beta T cell proliferation | *HLA-E,HLA-A* | 0.0299 |
| GO:2001187 | BP | 37 | positive regulation of CD8-positive, alpha-beta T cell activation | *HLA-E,HLA-A* | 0.0299 |
| GO:2000564 | BP | 37 | regulation of CD8-positive, alpha-beta T cell proliferation | *HLA-E,HLA-A* | 0.0299 |
| GO:2000566 | BP | 37 | positive regulation of CD8-positive, alpha-beta T cell proliferation | *HLA-E,HLA-A* | 0.015 |
| GO:0098602 | BP | 19 | single organism cell adhesion | *CD74,ACTB,B2M,ACTG1,S100A10,HLA-DRA,HLA-E,HLA-A,HLA-DPA1* | 0.00187 |
| GO:0016337 | BP | 19 | single organismal cell-cell adhesion | *CD74,ACTB,B2M,ACTG1,HLA-DRA,HLA-E,HLA-A,HLA-DPA1* | 0.0126 |
| GO:0034109 | BP | 19 | homotypic cell-cell adhesion | *CD74,ACTB,B2M,ACTG1,HLA-DRA,HLA-E,HLA-A,HLA-DPA1* | 0.001 |
| GO:0045785 | BP | 19 | positive regulation of cell adhesion | *CD74,S100A10,HLA-DRA,HLA-E,HLA-A,HLA-DPA1* | 0.0299 |
| GO:0022409 | BP | 19 | positive regulation of cell-cell adhesion | *CD74,HLA-DRA,HLA-E,HLA-A,HLA-DPA1* | 0.0475 |
| GO:0034112 | BP | 19 | positive regulation of homotypic cell-cell adhesion | *CD74,HLA-DRA,HLA-E,HLA-A,HLA-DPA1* | 0.022 |
| GO:1903039 | BP | 19 | positive regulation of leukocyte cell-cell adhesion | *CD74,HLA-DRA,HLA-E,HLA-A,HLA-DPA1* | 0.0226 |
| GO:0050870 | BP | 19 | positive regulation of T cell activation | *CD74,HLA-DRA,HLA-E,HLA-A,HLA-DPA1* | 0.0199 |
| GO:0051099 | BP | 9 | positive regulation of binding | *TXN,B2M,ANXA2,S100A10* | 0.0484 |
| GO:0002486 | BP | 7 | antigen processing and presentation of endogenous peptide antigen via MHC class I via ER pathway, TAP-independent | *HLA-A,HLA-B* | 0.015 |
| GO:0044419 | BP | 3 | interspecies interaction between organisms | *PSME1,RPL27A,B2M,RPL12,RPS4X,HLA-DRA,HLA-A,HLA-B* | 0.0481 |
| GO:0044403 | BP | 3 | symbiosis, encompassing mutualism through parasitism | *PSME1,RPL27A,B2M,RPL12,RPS4X,HLA-DRA,HLA-A,HLA-B* | 0.0481 |
| GO:0044764 | BP | 3 | multi-organism cellular process | *PSME1,RPL27A,B2M,RPL12,RPS4X,HLA-DRA,HLA-A,HLA-B* | 0.0292 |
| GO:0016032 | BP | 3 | viral process | *PSME1,RPL27A,B2M,RPL12,RPS4X,HLA-DRA,HLA-A,HLA-B* | 0.0261 |
| GO:0006457 | BP | 36 | protein folding | *CD74,ACTB,TXN,B2M,TUBA1A* | 0.0475 |
| GO:0032991 | CC | 29 | macromolecular complex | *CD74,ACTB,PSME1,EEF1A1,RPL36AL,RPL27A,B2M,TUBA1A,ANXA2,ACTG1,RPL12,RPS4X,HLA-DRA,HLA-E,HLA-H,HLA-A,HLA-DPA1,HLA-B* | 0.0254 |
| GO:0009986 | CC | 28 | cell surface | *CD74,B2M,ANXA2,HLA-DRA,HLA-E,HLA-H,HLA-A,HLA-DPA1,HLA-B* | 0.00127 |
| GO:0098797 | CC | 15 | plasma membrane protein complex | *B2M,HLA-DRA,HLA-E,HLA-H,HLA-A,HLA-DPA1,HLA-B* | 0.0109 |
| GO:0042611 | CC | 15 | MHC protein complex | *B2M,HLA-DRA,HLA-E,HLA-H,HLA-A,HLA-DPA1,HLA-B* | 7.55E-12 |
| GO:0042612 | CC | 15 | MHC class I protein complex | *B2M,HLA-E,HLA-H,HLA-A,HLA-B* | 2.85E-09 |
| GO:0022626 | CC | 24 | cytosolic ribosome | *RPL36AL,RPL27A,RPL12,RPS4X* | 0.0389 |
| GO:0005576 | CC | 6 | extracellular region | *CD74,ACTB,PSME1,CST3,NPC2,IGLL1,TXN,EEF1A1,B2M,TUBA1A,ANXA2,ACTG1,S100A4,POTEF,S100A10,RPL12,RPS4X,HLA-DRA,HLA-E,TMSB4X,HLA-A,HLA-B* | 2.83E-06 |
| GO:0044421 | CC | 6 | extracellular region part | *CD74,ACTB,PSME1,CST3,NPC2,IGLL1,TXN,EEF1A1,B2M,TUBA1A,ANXA2,ACTG1,S100A4,POTEF,S100A10,RPL12,RPS4X,HLA-DRA,HLA-E,HLA-A,HLA-B* | 9.25E-07 |
| GO:0043230 | CC | 6 | extracellular organelle | *CD74,ACTB,PSME1,CST3,NPC2,IGLL1,TXN,EEF1A1,B2M,TUBA1A,ANXA2,ACTG1,S100A4,POTEF,S100A10,RPL12,RPS4X,HLA-DRA,HLA-E,HLA-A,HLA-B* | 2.31E-09 |
| GO:0065010 | CC | 6 | extracellular membrane-bounded organelle | *CD74,ACTB,PSME1,CST3,NPC2,IGLL1,TXN,EEF1A1,B2M,TUBA1A,ANXA2,ACTG1,S100A4,POTEF,S100A10,RPL12,RPS4X,HLA-DRA,HLA-E,HLA-A,HLA-B* | 2.1E-09 |
| GO:0044444 | CC | 6 | cytoplasmic part | *CD74,ACTB,PSME1,CST3,NPC2,TXN,EEF1A1,RPL36AL,IFI27,RPL27A,B2M,TUBA1A,ANXA2,ACTG1,S100A4,POTEF,RPL12,RPS4X,HLA-DRA,HLA-E,TMSB4X,HLA-A,HLA-DPA1,HLA-B* | 0.00323 |
| GO:0031982 | CC | 6 | vesicle | *CD74,ACTB,PSME1,CST3,NPC2,IGLL1,TXN,EEF1A1,B2M,TUBA1A,ANXA2,ACTG1,S100A4,POTEF,S100A10,RPL12,RPS4X,HLA-DRA,HLA-E,TMSB4X,HLA-A,HLA-DPA1,HLA-B* | 1.85E-09 |
| GO:1903561 | CC | 6 | extracellular vesicle | *CD74,ACTB,PSME1,CST3,NPC2,IGLL1,TXN,EEF1A1,B2M,TUBA1A,ANXA2,ACTG1,S100A4,POTEF,S100A10,RPL12,RPS4X,HLA-DRA,HLA-E,HLA-A,HLA-B* | 2.31E-09 |
| GO:0044433 | CC | 6 | cytoplasmic vesicle part | *CD74,B2M,HLA-DRA,HLA-E,TMSB4X,HLA-A,HLA-DPA1,HLA-B* | 0.00172 |
| GO:0031988 | CC | 6 | membrane-bounded vesicle | *CD74,ACTB,PSME1,CST3,NPC2,IGLL1,TXN,EEF1A1,B2M,TUBA1A,ANXA2,ACTG1,S100A4,POTEF,S100A10,RPL12,RPS4X,HLA-DRA,HLA-E,TMSB4X,HLA-A,HLA-DPA1,HLA-B* | 8.82E-10 |
| GO:0012506 | CC | 6 | vesicle membrane | *CD74,B2M,HLA-DRA,HLA-E,HLA-A,HLA-DPA1,HLA-B* | 0.0048 |
| GO:0070062 | CC | 6 | extracellular exosome | *CD74,ACTB,PSME1,CST3,NPC2,IGLL1,TXN,EEF1A1,B2M,TUBA1A,ANXA2,ACTG1,S100A4,POTEF,S100A10,RPL12,RPS4X,HLA-DRA,HLA-E,HLA-A,HLA-B* | 2.1E-09 |
| GO:0005768 | CC | 6 | endosome | *CD74,CST3,B2M,TUBA1A,ANXA2,HLA-DRA,HLA-E,HLA-A,HLA-DPA1,HLA-B* | 0.000162 |
| GO:0044440 | CC | 6 | endosomal part | *B2M,ANXA2,HLA-DRA,HLA-E,HLA-A,HLA-DPA1,HLA-B* | 0.00365 |
| GO:0016023 | CC | 6 | cytoplasmic membrane-bounded vesicle | *CD74,B2M,ANXA2,HLA-DRA,HLA-E,TMSB4X,HLA-A,HLA-DPA1,HLA-B* | 0.0293 |
| GO:0030139 | CC | 6 | endocytic vesicle | *CD74,B2M,HLA-DRA,HLA-E,HLA-A,HLA-DPA1,HLA-B* | 7.25E-05 |
| GO:0045335 | CC | 6 | phagocytic vesicle | *B2M,HLA-E,HLA-A,HLA-B* | 0.0139 |
| GO:0030135 | CC | 6 | coated vesicle | *CD74,B2M,HLA-DRA,HLA-E,HLA-A,HLA-DPA1,HLA-B* | 2.12E-05 |
| GO:0030659 | CC | 6 | cytoplasmic vesicle membrane | *CD74,B2M,HLA-DRA,HLA-E,HLA-A,HLA-DPA1,HLA-B* | 0.00365 |
| GO:0030662 | CC | 6 | coated vesicle membrane | *CD74,B2M,HLA-DRA,HLA-E,HLA-A,HLA-DPA1,HLA-B* | 8.03E-07 |
| GO:0030133 | CC | 6 | transport vesicle | *CD74,B2M,HLA-DRA,HLA-E,HLA-A,HLA-DPA1,HLA-B* | 1.02E-05 |
| GO:0030134 | CC | 6 | ER to Golgi transport vesicle | *CD74,B2M,HLA-DRA,HLA-E,HLA-A,HLA-DPA1,HLA-B* | 1.32E-09 |
| GO:0030658 | CC | 6 | transport vesicle membrane | *CD74,B2M,HLA-DRA,HLA-E,HLA-A,HLA-DPA1,HLA-B* | 5.65E-08 |
| GO:0012507 | CC | 6 | ER to Golgi transport vesicle membrane | *CD74,B2M,HLA-DRA,HLA-E,HLA-A,HLA-DPA1,HLA-B* | 2.89E-10 |
| GO:0010008 | CC | 6 | endosome membrane | *B2M,ANXA2,HLA-DRA,HLA-E,HLA-A,HLA-DPA1,HLA-B* | 0.00243 |
| GO:0031901 | CC | 6 | early endosome membrane | *B2M,HLA-E,HLA-A,HLA-B* | 0.0434 |
| GO:0030666 | CC | 6 | endocytic vesicle membrane | *CD74,B2M,HLA-DRA,HLA-E,HLA-A,HLA-DPA1,HLA-B* | 1.86E-06 |
| GO:0030670 | CC | 6 | phagocytic vesicle membrane | *B2M,HLA-E,HLA-A,HLA-B* | 0.00271 |
| GO:0098552 | CC | 1 | side of membrane | *CD74,B2M,HLA-DRA,HLA-E,HLA-A,HLA-DPA1,HLA-B* | 0.00423 |
| GO:0098576 | CC | 1 | lumenal side of membrane | *CD74,HLA-DRA,HLA-E,HLA-A,HLA-DPA1,HLA-B* | 4E-09 |
| GO:0031300 | CC | 1 | intrinsic component of organelle membrane | *CD74,HLA-DRA,HLA-E,HLA-A,HLA-DPA1,HLA-B* | 0.00517 |
| GO:0031227 | CC | 1 | intrinsic component of endoplasmic reticulum membrane | *CD74,HLA-DRA,HLA-E,HLA-A,HLA-DPA1,HLA-B* | 6.03E-05 |
| GO:0098553 | CC | 1 | lumenal side of endoplasmic reticulum membrane | *CD74,HLA-DRA,HLA-E,HLA-A,HLA-DPA1,HLA-B* | 3.11E-09 |
| GO:0031301 | CC | 1 | integral component of organelle membrane | *CD74,HLA-DRA,HLA-E,HLA-A,HLA-DPA1,HLA-B* | 0.00396 |
| GO:0030176 | CC | 1 | integral component of endoplasmic reticulum membrane | *CD74,HLA-DRA,HLA-E,HLA-A,HLA-DPA1,HLA-B* | 4.73E-05 |
| GO:0071556 | CC | 1 | integral component of lumenal side of endoplasmic reticulum membrane | *CD74,HLA-DRA,HLA-E,HLA-A,HLA-DPA1,HLA-B* | 3.11E-09 |
| GO:0043209 | CC | 27 | myelin sheath | *ACTB,EEF1A1,TUBA1A,ANXA2,ACTG1* | 0.0104 |
| GO:0030881 | MF | 8 | beta-2-microglobulin binding | *HLA-E,HLA-H,HLA-A* | 0.000815 |
| GO:0003823 | MF | 10 | antigen binding | *CD74,HLA-DRA,HLA-E,HLA-H,HLA-A,HLA-DPA1,HLA-B* | 1.29E-07 |
| GO:0033218 | MF | 10 | amide binding | *CD74,CST3,HLA-DRA,HLA-E,HLA-H,HLA-A,HLA-DPA1,HLA-B* | 3.57E-06 |
| GO:0042277 | MF | 10 | peptide binding | *CD74,CST3,HLA-DRA,HLA-E,HLA-H,HLA-A,HLA-DPA1,HLA-B* | 1.56E-06 |
| GO:0042605 | MF | 10 | peptide antigen binding | *HLA-DRA,HLA-E,HLA-H,HLA-A,HLA-DPA1,HLA-B* | 6.4E-09 |
| CORUM:3055 | co | 26 | Nop56p-associated pre-rRNA complex | *EEF1A1,RPL27A,TUBA1A,RPL12* | 0.0485 |
| HP:0001339 | hp | 21 | Lissencephaly | *ACTB,TUBA1A,ACTG1* | 0.0469 |
| KEGG:05166 | ke | 17 | HTLV-I infection | *HLA-DRA,HLA-E,HLA-A,HLA-DPA1,HLA-B* | 0.0374 |
| KEGG:05130 | ke | 4 | Pathogenic Escherichia coli infection | *ACTB,TUBA1A,ACTG1* | 0.0268 |
| KEGG:04514 | ke | 38 | Cell adhesion molecules (CAMs) | *HLA-DRA,HLA-E,HLA-A,HLA-DPA1,HLA-B* | 0.00219 |
| KEGG:05169 | ke | 12 | Epstein-Barr virus infection | *HLA-DRA,HLA-E,HLA-A,HLA-DPA1,HLA-B* | 0.0113 |
| KEGG:05330 | ke | 18 | Allograft rejection | *HLA-DRA,HLA-E,HLA-A,HLA-DPA1,HLA-B* | 0.000002 |
| KEGG:03010 | ke | 2 | Ribosome | *RPL36AL,RPL27A,RPL12,RPS4X* | 0.0265 |
| KEGG:05332 | ke | 23 | Graft-versus-host disease | *HLA-DRA,HLA-E,HLA-A,HLA-DPA1,HLA-B* | 2.67E-06 |
| KEGG:04940 | ke | 35 | Type I diabetes mellitus | *HLA-DRA,HLA-E,HLA-A,HLA-DPA1,HLA-B* | 4.56E-06 |
| KEGG:04145 | ke | 14 | Phagosome | *ACTB,TUBA1A,ACTG1,HLA-DRA,HLA-E,HLA-A,HLA-DPA1,HLA-B* | 2.31E-07 |
| KEGG:05168 | ke | 22 | Herpes simplex infection | *CD74,HLA-DRA,HLA-E,HLA-A,HLA-DPA1,HLA-B* | 0.000484 |
| KEGG:05320 | ke | 33 | Autoimmune thyroid disease | *HLA-DRA,HLA-E,HLA-A,HLA-DPA1,HLA-B* | 1.27E-05 |
| KEGG:04612 | ke | 31 | Antigen processing and presentation | *CD74,PSME1,B2M,HLA-DRA,HLA-E,HLA-A,HLA-DPA1,HLA-B* | 4.18E-10 |
| KEGG:05416 | ke | 20 | Viral myocarditis | *ACTB,ACTG1,HLA-DRA,HLA-E,HLA-A,HLA-DPA1,HLA-B* | 6.2E-09 |
| MI:hsa-miR-33b | mi | 34 | MI:hsa-miR-33b | *CD74,TXN,EEF1A1,TUBA1A,RPS4X,TMSB4X* | 0.0137 |
| REAC:168256 | re | 25 | Immune System | *CD74,ACTB,PSME1,TXN,IFI27,B2M,ACTG1,HLA-DRA,HLA-E,HLA-A,HLA-DPA1,HLA-B* | 0.00873 |
| REAC:1280215 | re | 25 | Cytokine Signaling in Immune system | *PSME1,IFI27,B2M,HLA-DRA,HLA-E,HLA-A,HLA-DPA1,HLA-B* | 0.00789 |
| REAC:913531 | re | 25 | Interferon Signaling | *IFI27,B2M,HLA-DRA,HLA-E,HLA-A,HLA-DPA1,HLA-B* | 7.68E-05 |
| REAC:909733 | re | 25 | Interferon alpha/beta signaling | *IFI27,HLA-E,HLA-A,HLA-B* | 0.0054 |
| REAC:877300 | re | 25 | Interferon gamma signaling | *B2M,HLA-DRA,HLA-E,HLA-A,HLA-DPA1,HLA-B* | 1.68E-05 |
| REAC:983170 | re | 30 | Antigen Presentation: Folding, assembly and peptide loading of class I MHC | *B2M,HLA-E,HLA-A,HLA-B* | 7.59E-05 |
| REAC:156842 | re | 39 | Eukaryotic Translation Elongation | *EEF1A1,RPL27A,RPL12,RPS4X* | 0.0163 |
| REAC:156902 | re | 39 | Peptide chain elongation | *EEF1A1,RPL27A,RPL12,RPS4X* | 0.013 |
| REAC:1236975 | re | 16 | Antigen processing-Cross presentation | *PSME1,B2M,HLA-E,HLA-A,HLA-B* | 0.000398 |
| REAC:1236974 | re | 16 | ER-Phagosome pathway | *PSME1,B2M,HLA-E,HLA-A,HLA-B* | 0.000126 |
| REAC:1236977 | re | 16 | Endosomal/Vacuolar pathway | *B2M,HLA-E,HLA-A,HLA-B* | 2.41E-06 |

# Abbreviations: GO = Gene Ontology; BP = Biological Process; CC = Cellular Component; MF = Molecular Function; CORUM (CO) = Comprehensive Resource of Mammalian protein complexes; KEGG (KE) = Kyoto Encyclopedia of Genes and Genomes; HP = Human Phenotype Ontology; REAC (RE) = Reactome Pathway Database;

# ^a^The number of dysregulated genes found in the expression microarray data prevented functional enrichment analysis for other comparison groups.
